# A modulator based regulatory network for ERα signaling pathway

**DOI:** 10.1186/1471-2164-13-S6-S6

**Published:** 2012-10-26

**Authors:** Heng-Yi Wu, Pengyue Zheng, Guanglong Jiang, Yunlong Liu, Kenneth P Nephew, Tim HM Huang, Lang Li

**Affiliations:** 1 Center for Computational Biology and Bioinformatics, Indiana University, Indianapolis, IN, USA; 2 Indiana University School of Medicine, Indianapolis, IN, USA; 3 Medical Sciences, Indiana University School of Medicine, Bloomington, IN, USA; 4 Department of Molecular Virology, Immunology, and Medical Genetics, The Ohio State University, Columbus, OH, USA

## Abstract

**Background:**

Estrogens control multiple functions of hormone-responsive breast cancer cells. They regulate diverse physiological processes in various tissues through genomic and non-genomic mechanisms that result in activation or repression of gene expression. Transcription regulation upon estrogen stimulation is a critical biological process underlying the onset and progress of the majority of breast cancer. ERα requires distinct co-regulator or modulators for efficient transcriptional regulation, and they form a regulatory network. Knowing this regulatory network will enable systematic study of the effect of ERα on breast cancer.

**Methods:**

To investigate the regulatory network of ERα and discover novel modulators of ERα functions, we proposed an analytical method based on a linear regression model to identify translational modulators and their network relationships. In the network analysis, a group of specific modulator and target genes were selected according to the functionality of modulator and the ERα binding. Network formed from targets genes with ERα binding was called ERα genomic regulatory network; while network formed from targets genes without ERα binding was called ERα non-genomic regulatory network. Considering the active or repressive function of ERα, active or repressive function of a modulator, and agonist or antagonist effect of a modulator on ERα, the ERα/modulator/target relationships were categorized into 27 classes.

**Results:**

Using the gene expression data and ERα Chip-seq data from the MCF-7 cell line, the ERα genomic/non-genomic regulatory networks were built by merging ERα/ modulator/target triplets (*TF*, *M*, *T*), where *TF *refers to the ERα, M refers to the modulator, and T refers to the target. Comparing these two networks, ERα non-genomic network has lower FDR than the genomic network. In order to validate these two networks, the same network analysis was performed in the gene expression data from the ZR-75.1 cell. The network overlap analysis between two cancer cells showed 1% overlap for the ERα genomic regulatory network, but 4% overlap for the non-genomic regulatory network.

**Conclusions:**

We proposed a novel approach to infer the ERα/modulator/target relationships, and construct the genomic/non-genomic regulatory networks in two cancer cells. We found that the non-genomic regulatory network is more reliable than the genomic regulatory network.

## Background

Nuclear receptors (NR) are a superfamily of ligand-activated transcription factors that modulate specific gene expression by interacting with specific DNA sequence upstream of their target gene. So far there are over 100 nuclear receptors identified [1-3]. Estrogen receptor (ER) is a member of the nuclear receptor superfamily and is categorized into the class of ligand-dependent steroid receptor in the 1960s. The study explained it controls diverse biological processes by mediating the actions of steroid hormone estrogen and afforded an appreciation of its global importance in cell growth, cellular signalling, differentiation, maturation and homeostasis in eukaryotic cells. Finally, the general pathway for steroid hormone action was subsequently elucidated [4].

Unlike conventional transcription factors, ER is composed of several domains including ligand binding, DNA binding, dimerization, and transcriptional activation. The ligand binding domain participates in several activities including hormone binding, homo- and/or heterodimerization, and transcriptional activation and repression. The binding of the estrogen induces conformational changes in ER that could regulate gene expression by directed interaction with DNA (genomic pathway of ER action) or via an undirected connection with the modulation of some specific proteins (non-genomic pathway) [5,6].

In a gene regulatory network, gene transcription variations are controlled by many transcription factors. It has been established that the presence of regulatory sequences is in the proximity of genes and the existence of proteins is able to bind to those elements and to control the activity of genes by either activation or repression of transcription [7]. To understand gene regulation, the inference of its regulatory network is an important research topic [8]. Recent genomic technology, such as genome wide expression array or sequencing, allows us to elucidate the global gene regulatory mechanisms. Due to the well-developed microarray technology, the wealthy information for gene expression allows us to observe the expression levels of thousand of gene at once and helps more accurately predict gene-to-gene interaction according to its similarity or dissimilarity.

One approach to establish the gene regulatory network is to start from gene-gene correlations or interactions. Many computational approaches have been developed aimed to measure associations between mRNA abundant profiles to predict the transcriptional regulatory interaction. Some attempts at determining gene regulation based on the gene expression clustering algorithm. They group the genes that show similar gene expression using correlation coefficient matrix [9] or mutual information-based algorithm [10,11] under the same condition [8,9]. However, clustering the resembling genes that are co-regulated cannot present much more information about the biological mechanisms of gene regulation or regulatory pathway. Thus, some computational algorithms are proposed to reconstruct the gene networks by applying statistical approaches, such as Relevance Network, Bayesian Network, Linear Regression Network [12], and our own Regulation Network [3].

Relevance Network detects the relatedness between two genes from their gene expression profiles and gives a link between transcription factor and its target gene if correlated [13-17]. The typical methods to calculate the relatedness are Pearson Correlation Coefficient and Mutual information. Pearson Correlation Coefficient provides better performance on detecting linear relationships but it is not as intuitive as the Euclidean distance measure [17]. Mutual information (MI) gives good performance on non-linear relationship. For example, ARACNE algorithm [16] estimates the mutual information between the gene expressions of two genes using Gaussian kernel estimator. The measure of relatedness by MI ranges from 0 to 1. Relevance network is a relatively simple model, which computes the pair-wise similarity or dissimilarity between two genes.

Bayesian Network (BN) can identify casual relationships between variables. The topology a BN can provide the dependence or independence of variable [18], BN algorithm can reveal the dynamics of the gene regulation hierarchy. While BN has its advantage of structure model, it is difficult to inform whether a node (gene) is important to be included. Another challenging is its computational stability. It usually results in multiple optimal networks [19]. The high computational requirement leads to almost impossible of inference to a large-scale regulatory network [20]. Also, BN assumes no gene-gene interaction, which can misrepresent the data.

Our proposed ERα regulatory network is a combination of TF binding affinity estimated from ChIP-seq data, up or down regulation using gene expression, and motif conservation in probe sequences. This approach effectively utilized the genomic or non-genomic actions. Unlike previous regression approaches, this method did not use correlation information.

In this paper, our proposed approach analyzes the interaction between TF and target gene conditioned on a group of specific modulator genes. Also, we consider the change of modulators' expression level to perceive its influence on transcriptional activity. We reconstruct gene regulatory networks in related biological subjects via a *multiple linear regression *approach with interaction term such that the inferred modulator gene is directly embodied and the relationships of the biological subjects they represent are easily exploited. As a result, this reveals deeper insight on how the structure, function, and behaviour of components evolve.

## Method

### mRNA gene expression profiling data preprocessing

mRNA expression profiling for ERα was performed as previously described [2]. The microarray data for ERα in MCF-7 and ZR-75.1 cells treated with E2 were obtained from Experiment E-TABM-742 (http://www.ebi.ac.uk/arrayexpress/experiments/E-TABM-742). In this dataset, gene expression profile of human estrogen-responsivebreast cancer cell lines ZR-75.1 and MCF-7 treated with 10^-8 ^M of 17 β-estradiol (E2) on total RNA extracted before or after 1, 2, 4, 6, 8, 12, 16, 20, 24, 28 and 32 hours hormonal stimulation. The microarray data were preprocessed by BeadStudio Software version 3.2 with quantile normalization. Data were log2 transformed and the ratio of each signal against average reference signal were calculated [2]. There are 48702 probes in total.

More quality control analyses were performed. A probe is considered as absent if it is called absent in every time point by the BeadStudio Software, and these absent probes were excluded. Then, gene expression was averaged from the existing duplicate present probes. Genes with the small coefficient of variance are also removed. Using the threshold of 0.15, there were 6418 genes in MCF-7 cell and 13872 genes for ZR-75.1 cell left for the network analysis.

### ChIP-seq data analysis

ERα ChIP-seq data were prepared by Tim H. M. Huang's lab and generated for MCF-7 cell before and after E2 treatment 0.5, 1, and 24 hours. Sequencing was conducted with Illumina Genome Analyzer II (GA II) as per manufacturer's instructions. Reads are organized into a contiguous assembly of 24 different strands (one for each chromosome) and mapped to human genome reference sequence (HG18) using ELAND provided by Illumina. Four published peak-calling algorithms were applied to call the ERα binding sites: including CisGenome [21], GeneTrack [22], MACS [23], and SISSRS [24] with FDR = 0.001 to predict ERα binding peaks.

### Linear regression model with interaction term

A linear regression model (1), with an interaction term is constructed to describe the relationship among TF (ERα), M (modulator), and T (target).

(1) $$T={\text{a}}_{1}+{\text{a}}_{2}M+{\text{b}}_{1}TF+{\text{b}}_{2}TF\times M+\varepsilon ,$$

where (TF, T) are all J×1 vectors gene expression, and J is the total sampling time of gene expression samples; M is a binary variable (0 means low, and 1 means high) derived by the expression of a modulator's expression divided by its median; (*a_1_*, *a_2_*, *b_1_*, *b_2_*) are regression coefficients. For parameter setting, we did try the continuous scale for M at the beginning, and found that it often times it was highly sensitive to its skewed distribution because of small sample size. Therefore, a binary M is more robust choice. When the expression of modulator is high (*M *= 1), Eq. (1) becomes *T *= (a_1 _+ a_2_) + (b_1 _+ b_2_)*TF*; otherwise, *T *= a_1 _+b_1_*TF *when *M *= 0. Parameter *b*_2 _presents the change of T in response to *TF *influenced by *M*. If *b*_2 _is not statistically significant, the data do not have enough evidence to support M modulates the TF's effect on the target T.

### Schematic representation

Using a linear regression model with the interaction term, the regression coefficients (a_2_, b_1_, b_2_) define the functional relationship of the triplicate (T, TF, M). Eq. (2) defines the correlation indicator for all three regression parameters for any triplicate data analysis. The significant means p-value is less than 0.05.

(2) $$Correlation\;Indicator\left(CI\right)=\left\{\begin{array}{cc} +,\; & significant\;with\;positive\;value\\ -,\; & significant\;with\;negative\;value\\ 0,\; & not\;significant \end{array}\right)$$

**Figure 1:**
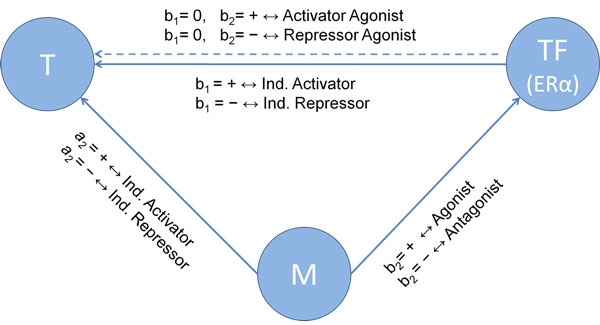
**Linear regression model**. A schematic overview of the triplicate network: Based on the criterion of correlation indicator (CI) described above, there are 27 categories (TF/M/T relationships) of network behaviours from all combinations.

A schematic overview of the triplicate network is shown in Figure 1. There are two lines between *TF *and *T*. These two lines mean two types of network construction according to the rule of modulator in the network. The solid line stands for the direct influence on the relationship between *TF *and *T*, which is independent of M; while the dashed line represents that the relationship of TF on T depends on M. Based on the criterion of correlation indicator (CI) described above, there are 27 categories of network behaviours from all combinations.

### Modulator gene candidates

We focus our modulators on specific molecular functional classes. Using Gene Ontology (GO) molecular function, several functional classes are included: protein kinase activity, phosphoprotein phosphatase activity, acetyltransferase activity, deacetylase activity, methltransferase activity, transcription factors, and transcriptional cofactors. In these 7 specific GO molecular functions, 485 unique modulator genes are found from the pool of 6418 presented genes in MCF-7 cell. The result of GO analysis is shown in Table 1.

**Table 1:** Gene ontology table for modulator genes

	GO:ID	# of unique modulator
**Signalling protein**		
Protein kinase activity	GO:0004672	162
Phosphoprotein phosphatase activity	GO:0004721	43
Acetyltransferase activity	GO:0016407	8
Deacetylase activity	GO:0019213	1
Methltransferase activity	GO:0008168	57

**TF protein**		
Transcription factor activity	GO:0030528	156
Transcription cofactor activity	GO:0003712	58

**Total**		485

### ERα genomic and non-genomic target gene selection

Using ChIP-seq analysis, only genes that have ERα bindings at 0, 0.5, 1, and 24 hours are considered genomic target. Four peak finding algorithms are used in predicting the binding sites: CisGenome, GeneTrack, MACS, and SISSRS with FDR = 0.001. Motif Conservation Score (MCS) [25] of each binding site overlapping with the known genes from the 6418 gene profiles is calculated, and a 95% conservation score is chosen as the threshold. As a consequence, 56 genes appearing in the results of 4 diverse algorithms can regard as the reliable target gene candidates in ERα genomic network. For the non-genomic network, we didn't treat all the other genes as non-genomic targets. As a matter of fact, only genes who don't have any binding signals with all the ChIP-seq peak finding tools in all four time points were selected as the non-genomic targets. There are 5877 genes not appearing in the results of 4 diverse algorithms.

### Network construction

Using the triplicates generated from the linear model with the interaction, a network model can be constructed. This initial network will include an enormous amount of modulators which have only limited targets. Therefore, a filtering threshold is developed to keep only modulators with significant number of targets. In this network analysis, all the connections among the modulators, targets, and the TF are assumed at random, though the total number of connections is fixed as the observed number from our previous analysis. After 1000 times shuffling in the network connections, a distribution of target number of any modulator is formed, and 95% threshold is chosen for the modulator selection.

### ERα regulatory network construction flowchart

**Figure 2:**
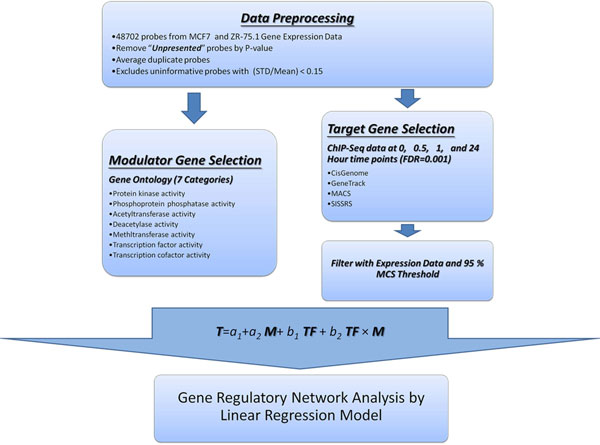
**The analytic workflow of gene regulatory network analysis**. An integration of analytic workflow for the proposed method is proposed. GRN construction goes through data pre-processing, modulator selection, genomic target/non-target selection, linear regression with interaction, and network construction.

The ERα regulatory network construction goes through data pre-processing, modulator selection, genomic target/non-target selection, linear regression with interaction, and network construction. Figure 2 shows an integration of analytic workflow for the proposed method.

## Results

### ERα/modulator categories

Using ChIP-seq data, we predicted 56 *ERα *genomic targets and 5877 non-genomic targets. Together with our pre-specified modulators, we generated 22 (T, TF, M) categories that involve the interactions with modulator genes. Many categories have FDR < 10% in both genomic and non-genomic (T, TF, M) categories. In particularly, genomic regulatory categories have higher FDRs than the non-genomic categories.

**Figure 3:**
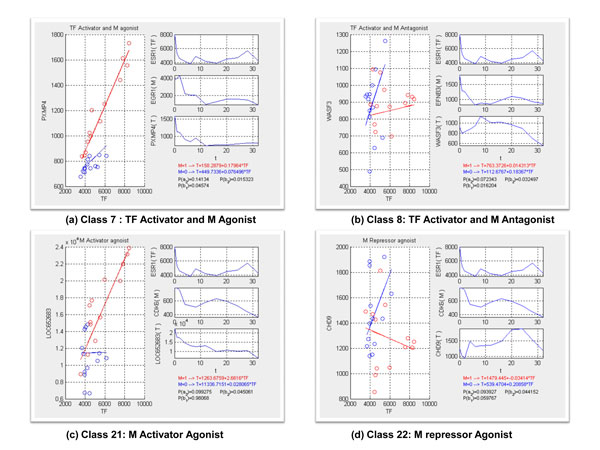
**Examples of the behavior of gene interaction**. 4 highlight examples of inferred gene interactions. (a) and (b) shows the modulator genes mediated the transcription activities, which enhance and repress the expression level of their corresponding target genes. (c) and (d) show CDK6 possesses the indirect influence on target genes according to the diverse level of its gene expression, which stimulate the TF activity.

The following are four highlighted examples. Figure 3(a,b) show the modulator genes mediated the transcription activities, which enhance and repress the expression level of their corresponding target genes. Figure 3(c,d) illustrates that CDK6 possesses the indirect influence on target genes according to the diverse level of its gene expression. Figure 3(c) represents CDK6 is inferred as an activator agonist of TF (ERα). Conventionally, ERα has no function on target gene (LOC652683). When the expression level of CDK6 comes to high, it stimulates ERα to turn into an activator to target gene (LOC652683). On the contrary, CDK6 owns the opposite capability to ERα since TF which makes no impact on target gene (CHD9) when CDK6 expression is high, which are illustrated in the Figure 3(d). It is implicated that each modulator gene can stimulate TF to either activate or repress a large number of targets, depending on the cellular context.

### ERα regulatory network analysis

Once a biological process is represented by a network, the analysis of network topology uncovers the functional organization and unknown organizing principles of cellular systems [26]. Many network researches investigate network activity for an active node by using the concept of degrees, defined as the number of edges adjacent to the neighbours. As a node has larger or higher connectivities, it represents to be the important connector and has more impact on the signalling pathway. For this reason, we zoom in our scope into a small-scale ERα regulatory networks.

**Figure 4:**
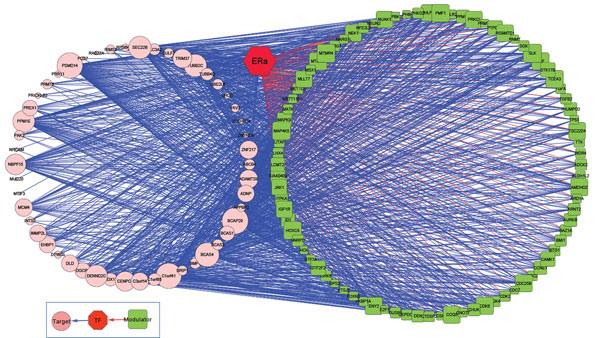
**ERα genomic network**. 85 selected modulator and 56 target genes constructed the ERα genomic network in MCF-7 cell.

**Figure 5:**
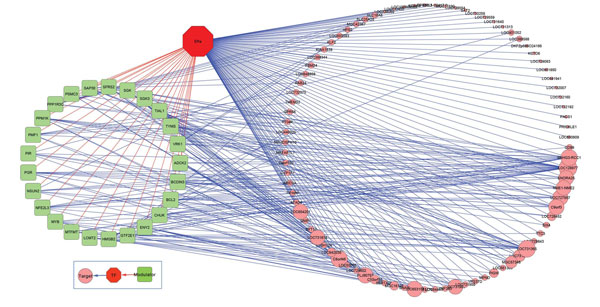
**ERα non-genomic network**. 25 selected modulator and 87 target genes constructed the ERα non-genomic network in MCF-7 cell.

To concentrate on those modulators with a large number of targets, ERα regulatory networks are constructed from the triplicates from Table 2. The thresholds on both MCF-7 and ZR-75.1cell were chosen based on the data itself, not on heuristics. A threshold of 18 targets for MCF-7 cell was chosen for modulator selection with a 0.1 level of FDR. Figure 4 and 5 visualize the ERα genomic and non-genomic network. ERα genomic regulatory network comprises of 85 modulators and 56 target genes. On the other hand, there are 25 modulators and 87 target genes in ERα non-genomic network. The gene marked with green rectangle represents modulator gene and the gene with pink circle is target gene. The size of rectangle or circle differentiates the degree of connections. The red colour line stands for the interaction between modulator and transcription factor, and blue line means the transcription activity between a pair of genes.

**Table 2:** Statistical results for modulator-TF-target Interaction

					ERα genomic			ERα non-genomic		
					
	Classification	a_2_	b_1_	b_2_	# of unique M/T	# of connection	FDR (%)	# of unique M/T	# of connection	FDR (%)
**1**	**TF (Ind. Activator)**	0	+	0	-	-	-	-	-	-
**2**	**TF (Ind. Repressor)**	0	-	0	-	-	-	-	-	-
**3**	**TF (Ind. Activator) and M (Ind. Activator)**	+	+	0	58/21	67	34.870	367/2590	6447	1.906
**4**	**TF (Ind. Repressor) and M (Ind. Activator)**	+	-	0	0/0	0	NA	0/0	0	NA
**5**	**TF (Ind. Activator) and M (Ind. Repressor)**	-	+	0	36/14	40	16.688	306/1035	3230	~ 0
**6**	**TF (Ind. Repressor) and M (Ind. Repressor)**	-	-	0	0/0	0	NA	144/88	349	~ 0
**7**	**TF (Activator) and M (Agonist)**	0	+	+	185/21	307	14.133	419/2372	71465	0.587
**8**	**TF (Activator) and M (Antagonist)**	0	+	-	282/47	908	3.676	470/4875	71166	0.824
**9**	**TF (Repressor) and M (Antagonist)**	0	-	+	94/2	96	0	258/206	5125	1.289
**10**	**TF (Repressor) and M (Agonist)**	0	-	-	0/0	0	NA	0/0	0	NA
**11**	**TF (Activator) and M (Agonist & Ind. Activator)**	+	+	+	0/0	0	NA	0/0	0	NA
**12**	**TF (Activator) and M (Antagonist & Ind. Activator)**	+	+	-	284/51	849	1.572	477/5204	78213	0.206
**13**	**TF (Repressor) and M (Antagonist & Ind. Activator)**	+	-	+	0/0	0	NA	0/0	0	NA
**14**	**TF (Repressor) and M (Agonist & Ind. Activator)**	+	-	-	0/0	0	NA	0/0	0	NA
**15**	**TF (Activator) and M (Agonist & Ind. Repressor)**	-	+	+	272/22	934	1.072	432/2171	51064	0.244
**16**	**TF (Activator) and M (Antagonist & Ind. Repressor)**	-	+	-	0/0	0	NA	0/0	0	NA
**17**	**TF (Repressor) and M (Antagonist & Ind. Repressor)**	-	-	+	23/2	26	12.837	332/442	8152	~ 0
**18**	**TF (Repressor) and M (Agonist & Ind. Repressor)**	-	-	-	0/0	0	NA	0/0	0	NA
**19**	**M (Ind. Activator)**	+	0	0	-	-	-	-	-	-
**20**	**M (Ind. Repressor)**	-	0	0	-	-	-	-	-	-
**21**	**M (Activator Agonist)**	0	0	+	261/37	835	4.797	418/3619	118241	0.479
**22**	**M (Repressor Agonist)**	0	0	-	42/8	65	15.404	213/977	5546	1.709
**23**	**M (Activator Agonist & Ind. Activator)**	+	0	+	0/0	0	NA	0/0	0	NA
**24**	**M (Repressor Agonist & Ind. Activator)**	+	0	-	23/9	34	19.633	354/1453	6119	0.951
**25**	**M (Activator Agonist & Ind. Repressor)**	-	0	+	277/40	638	2.093	440/3852	80644	0.336
**26**	**M (Repressor Agonist & Ind. Repressor)**	-	0	-	0/0	0	NA	0/0	0	NA
**27**	**No Function**	0	0	0	-	-	-	-	-	-

### Validation studies with ZR-75.1

**Figure 6:**
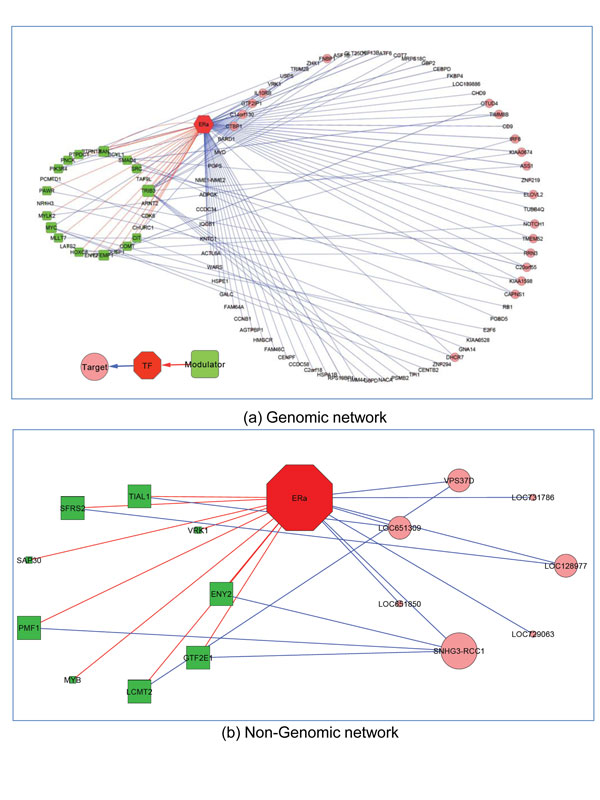
**Overlapping network between MCF-7 and ZR-75.1 cells**. (a) shows the overlapping genomic network between two cell lines. It consists 26 modulator and 71 target genes. (b) is the overlapping non-genomic network.

In the previous studies [27,28], both ERα-positive, hormone-responsive MCF-7 and ZR-75.1 cell had been well established to investigate the molecular and genomic mechanisms mediating hormonal control of cell function *in vitro*. Evidences in literature showed that some known genes in MCF-7 and ZR-75.1 cells are identified to have identical kinetic and type of response to hormone in breast cancer model after E2 stimulation. To investigate the target genes whose expression profile were significantly modified and the modulator genes who mediate the transcription activities of ERα action, gene expression data of ZR-75.1 was applied to our regulatory model to validate the results of MCF-7. It yielded (47/4799) overlapped triplets for genomic network and (12/270) triplets for non-genomic network, where 4799 and 270 are the number of triplets from the results of MCF-7. Using these overlapping triplets, ERα genomic/nongenomic network are exhibited in Figure 6.

For the results of the overlapping networks, the statistical significance of comparing the ERa network between two cancer cells is not our primary interests. As we have shown in our previous work, the overlap between the two gene regulatory networks in two different time points after E2 stimulation was very small even in a single cell line. We don't expect a significant overlap between two networks between two cells. The simply want to know what are the overlapped components and non-overlapped components. This description itself is very valuable. In addition, both GO and KEGG don't have estrogen signalling pathways, and we don't feel these analyses will add much to our understanding of the estrogen regulatory network.

## Conclusions

This paper proposed a regression model based approach in ERα regulatory network model construction. It characterizes the interaction between ERα and its modulators and their associated gene targets. With additional ERα binding information from ChIP-seq data, we are able to construct ERα genomic and non-genomic regulatory models. Comparing these two networks, ERα non-genomic network has lower FDR than the genomic network. This was validated by the same network analysis on both ZR-75.1 and MCF-7 cells.

## Competing interests

The authors declare that they have no competing interests.

## Authors' contributions

HYW designed the method and drafted the manuscript along with LL. PZ and GJ dealt with data processing. LL also critically revised the manuscript for important intellectual content. YL, KPN, THMH and LL supervised the work and gave final approval of the version of the manuscript to be submitted.
